# Using resting state functional MRI to build a personalized autism diagnosis system

**DOI:** 10.1371/journal.pone.0206351

**Published:** 2018-10-31

**Authors:** Omar Dekhil, Hassan Hajjdiab, Ahmed Shalaby, Mohamed T. Ali, Babajide Ayinde, Andy Switala, Aliaa Elshamekh, Mohamed Ghazal, Robert Keynton, Gregory Barnes, Ayman El-Baz

**Affiliations:** 1 Bioimaging Lab, Bioengineering Department, University of Louisville, Louisville, KY, United States of America; 2 Department of Electrical and Computer Engineering, Abu Dhabi University, Abu Dhabi, United Arab Emirates; 3 Department of Neurology, University of Louisville, Louisville, KY, United States of America; 4 Bioengineering Department, University of Louisville, Louisville, KY, United States of America; Yale University, UNITED STATES

## Abstract

Autism spectrum disorder (ASD) is a neuro-developmental disorder associated with social impairments, communication difficulties, and restricted and repetitive behaviors. Yet, there is no confirmed cause identified for ASD. Studying the functional connectivity of the brain is an emerging technique used in diagnosing and understanding ASD. In this study, we obtained the resting state functional MRI data of 283 subjects from the National Database of Autism Research (NDAR). An automated autism diagnosis system was built using the data from NDAR. The proposed system is machine learning based. Power spectral densities (PSDs) of time courses corresponding to the spatial activation areas are used as input features, feeds them to a stacked autoencoder then builds a classifier using probabilistic support vector machines. Over the used dataset, around 90% of sensitivity, specificity and accuracy was achieved by our machine learning system. Moreover, the system generalization ability was checked over two different prevalence values, one for the general population and the other for the of high risk population, and the system proved to be very generalizable, especially among the population of high risk. The proposed system generates a full personalized report for each subject, along with identifying the global differences between ASD and typically developed (TD) subjects and its ability to diagnose autism. It shows the impacted areas and the severity of implications. From the clinical aspect, this report is considered very valuable as it helps in both predicting and understanding behavior of autistic subjects. Moreover, it helps in designing a plan for personalized treatment per each individual subject. The proposed work is taking a step towards achieving personalized medicine in autism which is the ultimate goal of our group’s research efforts in this area.

## Introduction

Autism spectrum disorder (ASD) is a neuro-developmental disorder associated with three main characteristics [1]: (i) impairments in social functioning, (ii) communication difficulties, and (iii) restricted and repetitive behaviors. The wide variation of clinical and genetic heterogeneity between autistic subjects [2] has made precision medicine a trending approach for diagnosis and treatment. Precision medicine combines both pathophysiologically based treatments and objective biomarkers to predict the most beneficial treatment for a particular subject. For drugs currently in clinical trials, investigation of the relationships between response and etiologies/biomarkers should be explored to better understand individualized effects for development of subsequent larger trials. The goal would be to optimize targeted treatment for patients with ASD. Existing collaborative approaches of this type have not yet targeted environmental etiologies/risk factors. As our understanding of environmental factors expands, it will be critical to incorporate these factors into experimental approaches. Although ASD is a heterogeneous disorder which varies in both symptoms and severity for each subject, it can be systematically assessed utilizing a data driven approach to split ASD into subgroups. Each subgroup can be explored separately to develop individualized/personalized treatments. The proposed approach will be more efficacious to optimize ASD treatment for each subject rather than conventional methods that are applied broadly for all ASD subjects. Personalized interventions at early ages may show a profound effect on ASD subjects during development. In combination with impactful behavioral therapies (such as early intensive behavioral intervention), this approach will have a significant impact on the overall symptoms of ASD over a lifetime. Consequently, this study has two main objectives: (i) design and implement an accurate machine learning system to classify ASD and TD correctly, and (ii) provide a personalized map that shows the affected areas and severity of autism for each ASD subject. Accomplishing these objectives will facilitate the designing of a precise personalized plan for each autistic subject.

Connectivity analysis is a very common way to determine the abnormalities between ASD and TD subjects [3, 4], where three major patterns are analyzed: (i) gray matter structural connectivity, (ii) white matter structural connectivity, and (iii) functional connectivity. Within the gray matter, microstructural abnormalities in autistic subjects reported, for example, [5] and [6], are suggestive of corresponding changes in connectivity. There have been few direct studies of synaptic connections in autism using human postmortem tissue [7], and alterations in grey matter connectivity have mostly been inferred from other findings. The minicolumns, basic anatomical and functional units of the cerebral cortex, have been found to be more narrow and/or more numerous in autism [5, 6]. The reduction in neuropil around these minicolumns in particular has implications for connectivity. It may lead, for example, to a reduction in the inhibitory capacity of GABAergic interneurons [8]. Other evidence of disrupted synaptic connectivity derives from genetic studies, which have linked mutations in proteins involved in synaptic transmission with the incidence of autism. Studies in mouse models have elucidated what impact these mutations might have in the human brain [7, 9]. Another connectivity abnormality which was detected in the white matter of autistic subjects is the reduced long range connectivity and increased short and medium range connectivity [10]. The reduced long range connectivity was expressed in terms of reduced fractional anisotropy in autistic subjects in many recent studies, for example [11]. Functional connectivity analysis is the third type of analysis. Each subject is asked to perform a task or stay at rest without falling asleep in order to apply a functional connectivity analysis on his/her brain scan. [12]. Underconnectivity theory [13] states that both neurobiological and cognitive disorders are the main causes of ASD. Synchronized brain activity reduction in integrative processing demanding tasks, such as forming a sentence from 2 or more words, is used to depict the cognitive disorder. More recent studies investigated the brain connectivity associated with different tasks. For example, less activation in the left dorsolateral prefrontal and inferior parietal areas was identified, while more activation was recorded in the right occipital (visuospatial) areas and bilateral superior parietal regions were reported in a figures task experiment in [14]. In [15], the response to facial expressions was addressed, where autistic individuals showed higher activation in the amygdala, ventral prefrontal cortex, and striatum specifically for sad facial expressions. Another type of task based experiments is the rewards task, where subjects are given either monetary or social reward and their brain activity in response to this reward is recorded [16, 17]. In [18], less activation in the right nucleus accumbens and more activation in left midfrontal and anterior cingulate gyrus were reported in autistic subjects than were reported in healthy controls in response to social and monetary rewards. Another study [19] revealed less connectivity in autistic subjects in response to rewards. Also, in [20], a machine learning algorithm (multivariate autoregressive model) was used to study the alternation in connectivity between the two groups while trying to find the most logical end to a story shown to them.

Resting state is another method used to study the brain activity without performing any task. Resting state brain connectivity has been discussed in various studies. The underconnectivity hypothesis was supported by [21], where less functional brain connectivity was found in autistic subjects than that found in healthy control subjects. This result was supported for autistic males by [22], while autistic females and autistic children showed hyperconnectivity in [23]. In [23], autistic children with more severe social dysfunction were found to be functionally hyperconnected. In [24], decreased connectivity was noticed in local areas in the frontal and temporal cortex, but no global abnormalities were detected. Also, in [25], reduced connectivity in visuospatial and superior parietal areas was reported in autistic subjects as compared to healthy control subjects. Reduced connectivity was also reported in [26] in both dentate nucleus and cerebello-thalamo-cortical (CTC) circuits. Building on previous studies, [27] depicted alternations in connectivity patterns. These alternations in connectivity patterns appeared in the interhemispheric connectivity analysis, where for autistic subjects there have been areas with decreased connectivity and other areas with increased connectivity compared to the same areas in TD subjects. The altered connectivity result was also supported by [28], where both hypoconnectivity and hyperconnectivity were reported in autistic subjects. Another study [29], reported dysfunction in the functional networks, and this dysfunction was more obvious in social information processing related networks.

In addition to its importance in reporting group differences between healthy controls and implicated subjects, studying the resting state connectivity patterns showed promising results in diagnosis of many diseases such as schizophrenia [30], Alzheimer’s disease, [31] and autism. In [30], a deep neural network was used for whole brain classification of schizophrenia. The approach in [30] achieved high accuracy in schizophrenia diagnosis. In autism diagnosis, a recent study in [32] used deep neural network to build a diagnostic system using functional connectivity correlation matrix as input to the network.

Many of the mentioned studies, and others [33], reported findings in different brain areas or lobes among the autistic subjects and healthy controls. This helps in understanding autism causative factors. But to the best of our knowledge, localized abnormalities for each subject haven’t been reported by any of the formerly mentioned studies. Due to the heterogeneity of autism and its various etiology and severity, a more personalized approach is needed to predict and analyze the affected behavior and functionality of each subject; hence, an optimal goal is to achieve an individually designed personalized treatment plan.

In this study, we are expanding our group’s previous work [34], where a resting state analysis is performed on a dataset with a relatively large number of subjects (283 subjects). The work flow of this analysis is to extract the features of most importance from the time courses corresponding to functional connectivity spatial maps of both autistic and healthy control subjects. Using the extracted features, we build a CAD system that is able to provide a global diagnosis decision for each subject; additionally, it provides a local diagnosis report that shows the most affected areas in the brain, which could help in better understanding and predicting of the affected behaviors and functionalities for each individual subject. In addition to increasing the number of subjects, we provide a correlation analysis between the CAD system output and the Autism Diagnosis Observation Schedule ADOS behavioral reports. This analysis reflects how the CAD system is able to predict the affected behaviors and allows for as early intervention as possible.

## Materials and methods

### Data description

In this study, we obtained fMRI data for 123 ASD and 160 TD children and adolescents (for a total number of 283 subjects) from the National Database for Autism Research (NDAR: http://ndar.nih.gov). Imaging data hosted by NDAR are fully anonymized and linked with other records (diagnostic, behavioral, demographic, etc.) via an opaque identifier, the NDAR globally unique identifier (GUID). GUIDs for all subjects used in this study are provided in the supplemental materials—S1 Table. The data used are obtained from two studies, one done at George Washington University (study ID 2021) and the other at UCLA Autism Center of Excellence (study ID 2026). We selected only subjects who have resting state fMRI, structural MRI, and DTI data available because our planned future work is to expand this work to be a multi-modal personalized diagnosis system. All of the participant subjects have both a high-resolution T1 weighted structural MRI and a resting state functional MRI (fMRI). In addition to the imaging data, many subjects also have (i) cognitive/behavioral data in the form of BRIEF-parent (100 autistic and 140 healthy controls), (ii) child/adolescent symptom inventory (CASI) (67 autistic and 110 healthy controls), (iii) child behavior checklist (CBCL) for ages 6–18 (116 autistic and 160 healthy controls), and (iv) differential ability scales 2nd edition (DAS-II) (105 autistic and 148 healthy controls). Those with a diagnosis of ASD usually had associated scores on the (v) ADOS reports (96 autistic) and (vi) Autism diagnostic interview (ADI-R) (117 autistic). Resting state fMRI and structural MRI data used in this study was acquired using Siemens Magnetom TrioTim with a 3 T magnet. Structural MRI data used an MPRAGE pulse sequence with TR = 2530 ms, TE = 3.31 ms, TI = 1100 ms, and flip angle 7°. Voxel spacing for structural MRI volumes is isotropic with 1 mm. Resting state fMRI scans have TR = 2000 ms, TE = 2000 ms, and flip angel 90° in a two dimensional acquisition sequence to produce images with 3 mm pixel spacing and 4 mm slice spacing. Time to acquire 33 coronal slices spanning the entire brain was 2.01 s, and the resting state data were recorded for approximately 6 min, as it was described in study ID 2021. While for study ID 2026, TR = 3000 ms, TE = 28 ms and flip angle 90° in a two dimensional acquisition sequence to produce images with 3 mm pixel spacing and 4 mm slice spacing. Time to acquire 34 coronal slices spanning the entire brain was 3.01 s, and the resting state data were recorded for approximately 6 minutes.

### Resting state fMRI experiment

The main objective while analyzing a resting state fMRI (R-fMRI) scan is to study the low-frequency fluctuations measured in blood oxygenation level dependent (BOLD) signal, which identify spatial and temporal characteristics of the resting state networks (RSNs) [35]. To localize the individual abnormalities, the RSNs of each subject are then mapped to four resting state standard brain atlases by checking the correlation between each atlas area and each RSN. The package used in this experiment for both analysis and preprocessing is FSL MELODIC (Multivariate Exploratory Linear Optimized Decomposition into Independent Components) [36].

#### R-fMRI preprocessing

In this study, we applied multiple preprocessing steps on R-fMRI scans before the analysis takes place:

- Brain Extraction Tool (BET) is used to skull stripping. Skull stripping is where we segment an MRI image into a brain and non brain. BET used deformable model for segmenting the brain and it is considered as a robust tool [37].
- Removing the time differences between acquired 2D slices of fMRI scans using slice timing correction in increasing order.
- To eliminate the effect of subject movement during MRI scan, we apply motion correction using MCFLIRT algorithm [38, 39].
- In order to increase signal to noise ration (SNR), and accommodates for the individual anatomical variations inter-subjects, we used Gaussian filtering for spatial smoothing. we used Gaussian filter with full width half maximum (FWHM) of 2 mm [40]. The relatively small FWHM was selected to avoid activation cluster merging [41].

With the preprocessed scans, we apply two-phase registration on each scan. First, we registered every preprocessed fMRI scan to it’s corresponding high resolution T1-weighted structural image. Second, we aligned these preprocessed registered fMRI scans to MNI152 standard space. For both registration steps, we used a 12 degree of freedon affine transformation. The registration is performed using FLIRT software in the FSL package.

#### R-fMRI data analysis and feature extraction

In R-fMRI, the values of BOLD signal at every voxel over time represents a signal that comprises spatial locations and their corresponding activation time courses. Functional connectivity is defined as the minimal loss decomposition of the source signal (BOLD signal values) into two independent components (spatial locations and time courses). The famous blind source separation problem (BSS) is somehow analogous to our source signal decomposition problem, at which we need to recover set of statistical independent sources signal from a measured signal that comprises a mixture of sources [42]. The BSS assumes that there is no prior knowledge about the sources or the mixture structures.

The BSS problem can be formulated as:

$$\begin{array}{c} x_{i}\left(t\right)=As_{i}\left(t\right)+\eta_{i}\left(t\right) \end{array}$$ (1)

Where *x*_*i*_ is the BOLD signal measured over time at voxel *i*, *s*_*i*_ is the non-Gaussian source signal, *η*_*i*_ ∼ *N*(0, *σ*^2^∑_*i*_), and *A* is the mixing matrix. To solve this BSS problem it is required to find the unmixing matrix *W* such that

$$\begin{array}{c} {\hat{s}}_{i}=Wx_{i} \end{array}$$ (2)

is a close approximation of the original measured signal. To solve this BSS problem in the presence of Gaussian noise, a probabilistic independent component analysis (PICA) algorithm is used. In the presence of unknown noise covariance, the unmixing matrix *W* is estimated in an iterative manner, by iterating estimates of the mixing matrix and the independent sources then reestimating the noise covariance from the residuals. For more mathematical details about finding the solution, uniqueness, correctness, and model order, the reader is referred to [36].

**Fig 1:**
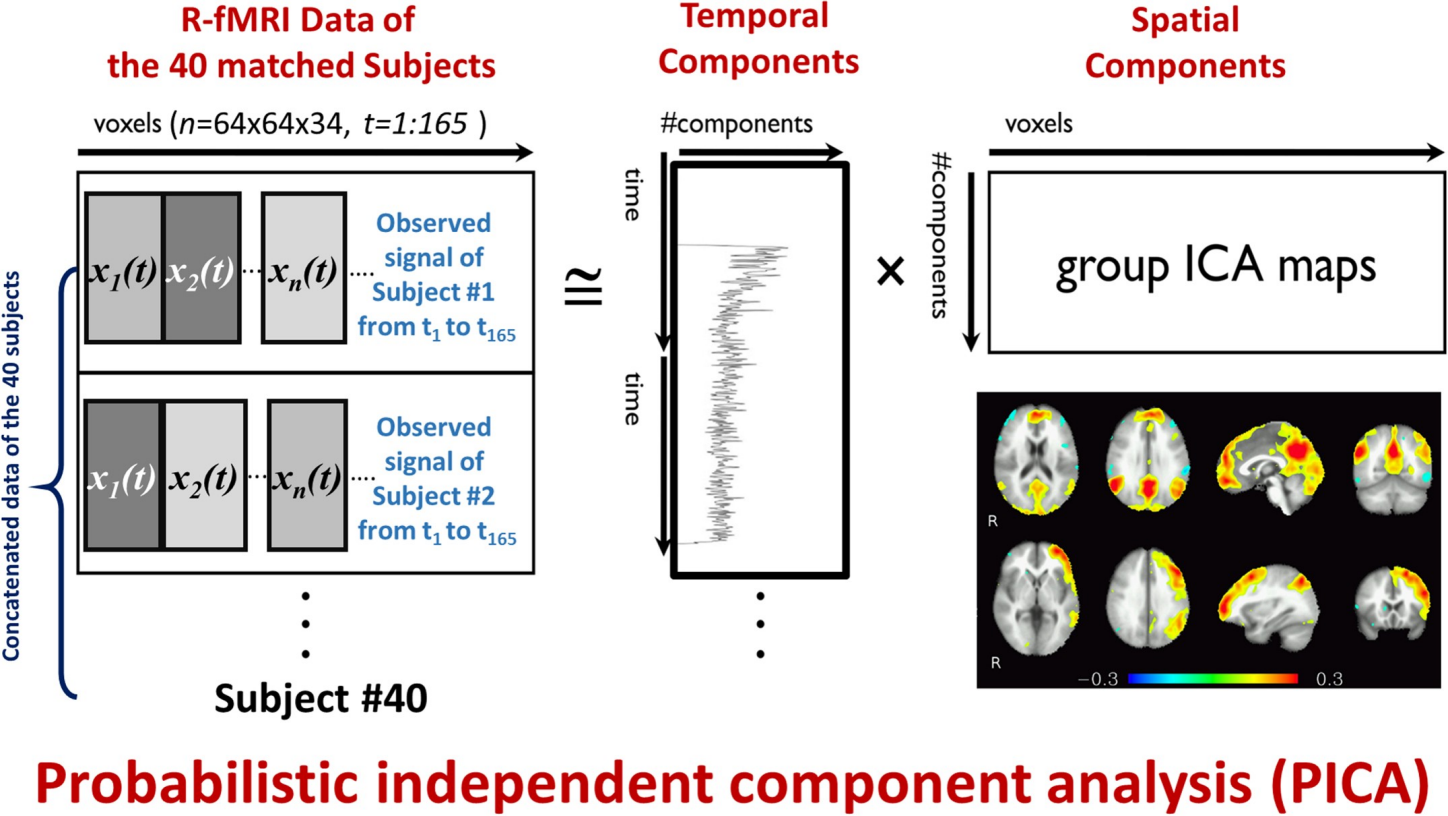
The PICA analysis of R-fMRI data, the observed vectors of voxel values (*x*_1_(*t*), *x*_2_(*t*), …*x*_*m*_(*t*)) over time *t* are the input, and they are decomposed into spatial components and their activation time courses.

In this study, 40 matched subjects (20 ASDs and 20 TDs) in terms of age, gender, and IQ are used for group ICA analysis, where subjects are temporally concatenated. The output of the group PICA is 34 spatial components that represent activation patterns in the 40 subjects. To assess statistical significance between the 2 groups, permutation testing and Bonferroni correction are applied to the output components. To obtain spatial components and time courses for each individual subject dual regression is applied. In the first regression phase, group spatial components are used with the subject 4D volume to obtain subject specific time course, then a second regression phase is applied to obtain subject spatial components using the time courses obtained in the first phase. The pipeline is illustrated in Fig 1.

**Fig 2:**
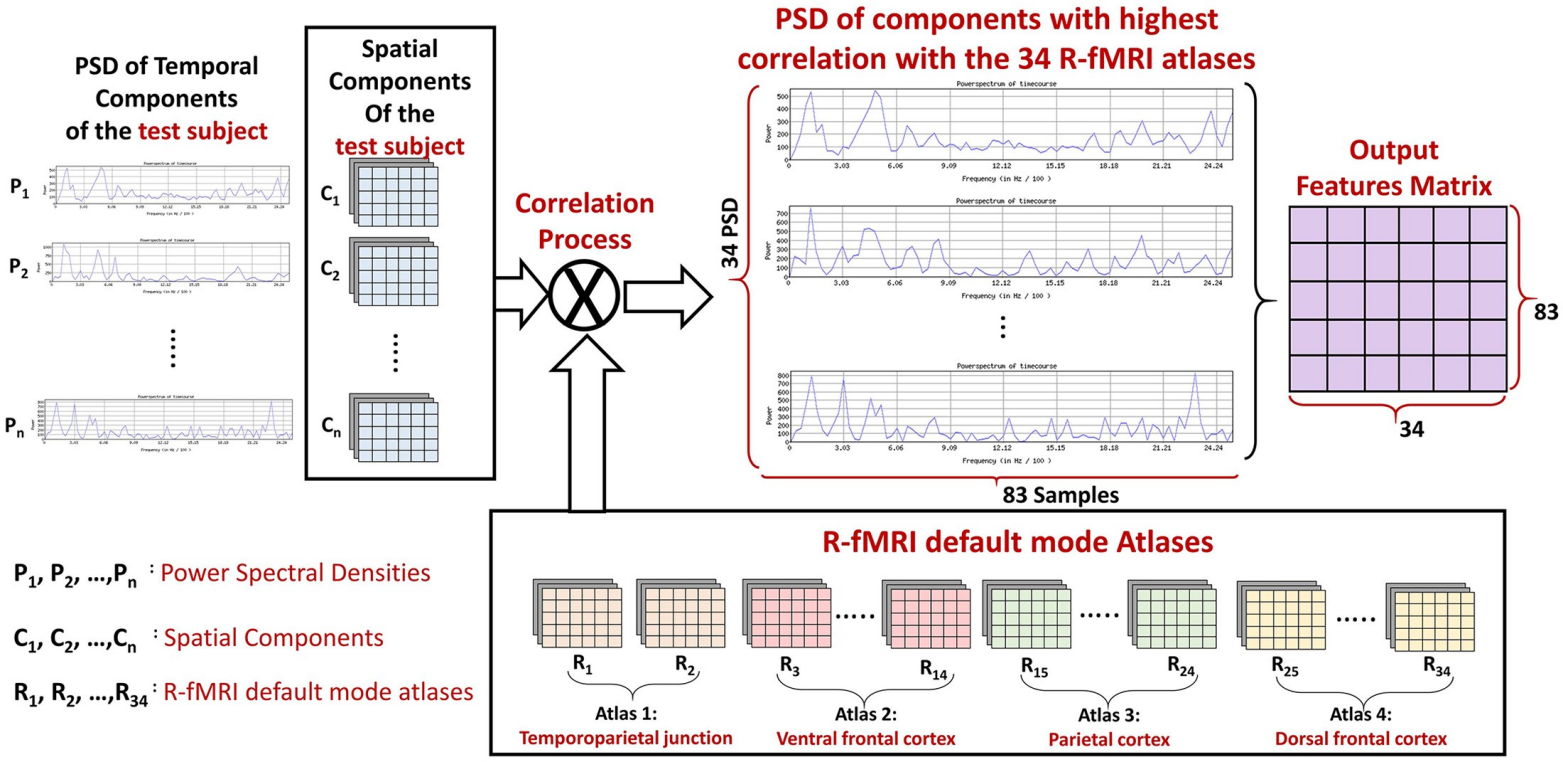
The correlation between the 34 components of the functional atlas and every output spatial map. PSDs of the time courses corresponding to the areas of interested are used as features.

After completing the PICA analysis, we used an atlas of 34 areas as a reference of connectivity networks. we calculated the correlation between every area of that atlas and every extracted spatial map. The area of interested are then selected based on those correlation values, such that areas with maximum correlation are selected. The features used in diagnosis are the power spectral densities (PSDs) corresponding to the activation time courses of the 34 areas of interest. PSDs were used as features because they represent a sensitive way for BOLD signal oscillations description which enhances the ability to analyze the network connectivity [43]. Also, PSD has another advantage, which is being time shift invariant. This means that, among different subjects, if the same activation happens but at different shifts in time, the PSD will not be affected. The feature extraction process is illustrated in Fig 2.

#### Resting state functional atlas

In this study, we used four local atlases to create an atlas that defines the expected activation networks during resting state. These four local atlases describe 34 different cortical areas, and those local atlases are:

1. Parietal cortex atlas [44]: In this atlas study, both functional connectivity and anatomical connectivity were studied on humans and macaques. Accordingly, the parietal cortex was divided into 10 components, 5 in the inferior parietal lobule (IPL) and 5 in the superior parietal lobule (SPL). Those components were clustered based on cross correlation in the tractography-based connectivity patterns of parietal voxels.
2. Temporoparietal junction (TPJ) atlas [45, 46]: In this atlas study, TPJ was examined to check if it is a single area with a heterogeneous functional connectivity or multiple areas, each with its unique connectivity pattern. Accordingly, TPJ was parcellated into 2 components: (i) anterior TPJ cluster, which showed interaction with ventral prefrontal cortex and anterior insula and (ii) posterior TPJ cluster which showed interaction with the posterior cingulate, temporal pole, and anterior medial prefrontal cortex.
3. Dorsal frontal cortex [47]: In this atlas study, both DTI and fMRI were used to compare the dorsal frontal cortex organization between humans and macaques. According to this study, the human dorsal frontal cortex is parcellated into 10 components. They are all between the human inferior frontal sulcus and the cingulate cortex.
4. Ventral frontal cortex [48]: In this atlas study, similarities and differences between human and macaques’ ventral frontal cortex were identified. Based on the study outcome, the ventral frontal cortex was divided into 11 components, in addition to one more component from the ventrolateral frontal pole.

**Fig 3:**
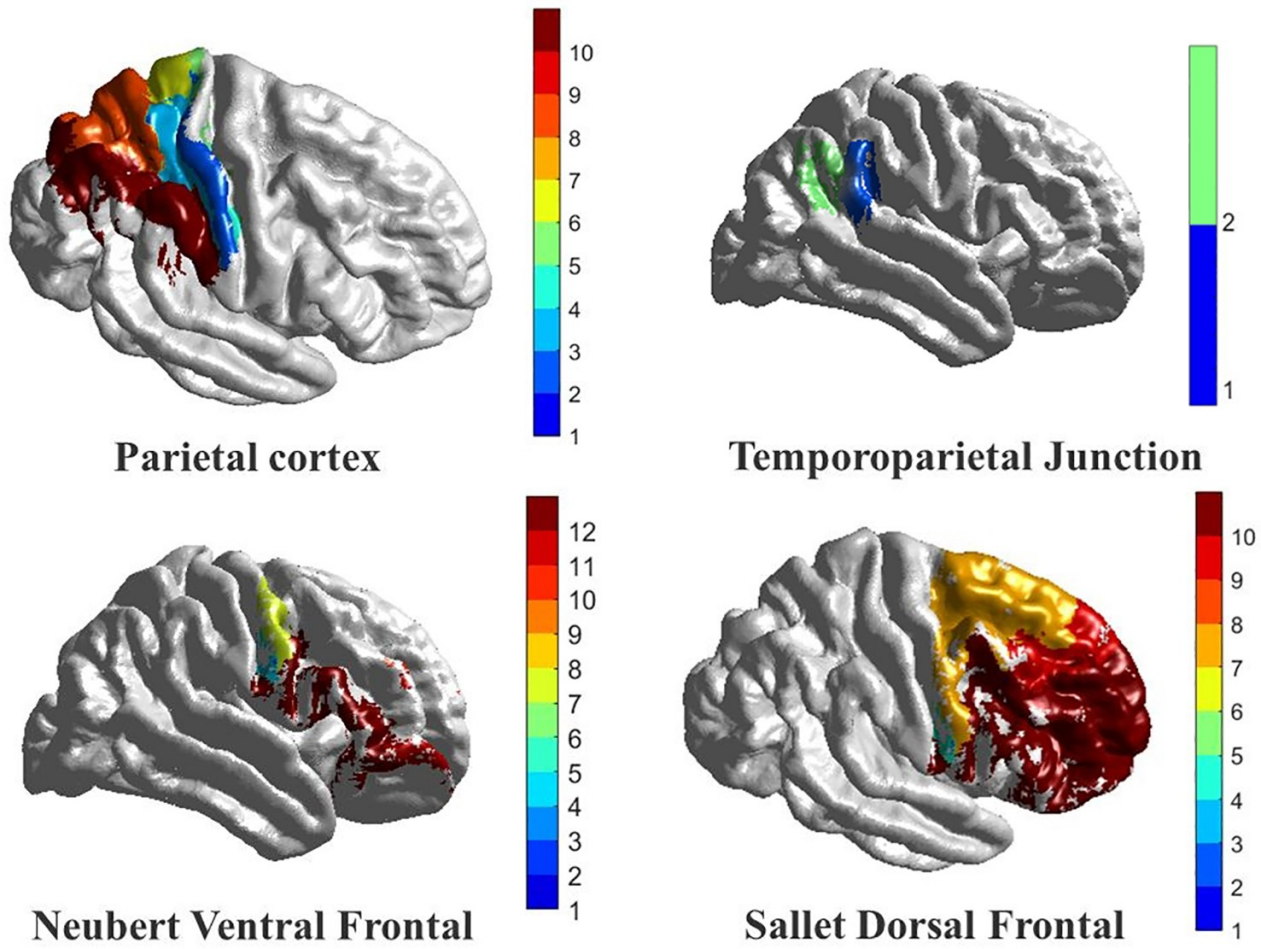
The four functional connectivity atlases used in this study, with a color coded map to show the 10 subareas in the parietal cortex, the 2 subareas in the temporoparietal junction, the 12 subareas in the ventral frontal lobe, and the 10 subareas in the dorsal frontal lobe.

More details about the functional atlas used and the components are illustrated in Table 1. Also the physical locations of the used atlases are illustrated in Fig 3.

**Table 1:** The components defined by the four functional atlases and the corresponding anatomical areas.

Atlas	Component	Anatomical area/region
Parietal cortex	SPLA	Ventral intraparietal area (BA 7)
	SPLB	BA 5
	SPLC	Antero-medial intraparietal sulcus
	SPLD	BA 7
	SPLE	Posterior intraparietal sulcus
	IPLA	Parietal operculum
	IPLB	Anterior supramarginal gyrus
	IPLC	Posterior supramarginal gyrus
	IPLD	Anterior angular gyrus
	IPLE	Posterior angular gyrus
TPJ	TPJa	Areas 39, 40, 22
	TPJp	Areas 39, 40, and 22
Neubert Ventral Frontal	6v	BA 6
	6r	BA 6
	IFJ in areas 6, 9, and 44	Inferior frontal junction (BA 6, 9, 44)
	44d	BA 44, dorsal
	44v	BA 44, ventral
	45A	BA 45
	45	BA 45
	47	BA 47
	IFS in area 9	Inferior frontal sulcus (BA 9)
	46	BA 46
	FPl in area 10	Lateral frontopolar region (BA 10)
	FPm in area 10	Medial frontopolar region (BA 10)
Sallet Dorsal Frontal	Cluster1	Supplemental motor area (BA 6)
	Cluster2	Pre-supplemental motor area (BA 6)
	Cluster3	BA 9
	Cluster4	BA 10
	Cluster5	BA 9, 46
	Cluster6	BA 9, 46
	Cluster7	BA 46
	Cluster8	BA 8
	Cluster9	Anterior, dorsal premotor area (BA 6)
	Cluster10	BA 8

### Local and global classification

To build our diagnostic system, which is expected to (i) classify/diagnose ASD and TD subjects, and (ii) identify local areas with autism related impairments, we used the extracted PSDs as our discriminating features between the ASD and TD groups. However, to enhance the classification process of our diagnostic system, we fed the classifier with a higher level representation of the PSDs.

We used 34 sparse autoencoders (SAEs), such that there is an autoencoder for each functional area, to represent our PSDs in a higher level representation and also to reduce the feature vectors dimensionality [49–52]. We used autoencoders to encode the PSDs through a set of nonlinear filters to a new space. Thus, when decoding them again, they give a reconstructed version of the input with minimal reconstruction error [53, 54]. In the training phase, SAEs weights were updated through error backpropagation with batch gradient descent, where the L-BFGS optimization algorithm [55] is used for reconstruction error minimization.

To find the optimal set of hyper-parameters for the SAEs network, typically the number of layers, number of nodes in each layer (*range*: 10: 100), sparsity parameter (*range*: 0.05: 0.9), sparseness control parameters (*range*: 1: 20), and L2 regularization (*range*: 10^−3^: 10^−6^), a grid search algorithm with the reconstruction error as the metric to optimize is used Supplemental materials—S1 Fig).

To show the effect of hyper-parameters fine tuning supplementary materials S2 Table showing different sets of hyper-parameters and the corresponding accuracies for each area is uploaded.

After extracting the high level features using SAEs, they are fed into a probabilistic support vector machine (SVM) classifier with RBF kernel to obtain posteriori class membership scores, where the class membership was calculated as the sigmoid of the distance between the sample and the classification hyperplane. The hyper-parameter of the SVM, typically the kernel scale (*range*: 1: 20) and box constraint (*range*: 1: 100), are also selected using grid search [56] using accuracy as the metric to optimize. The selected kernel scale and box-constraint are 5 and 12, respectively. For the global subject diagnosis, we propose a heuristic based on a winner-takes-all approach. All the significant areas scores are averaged per subject, and the class with the largest average value is considered the final global diagnosis for the corresponding subject.

Statistical significance of classifier accuracy was assessed using bootstrapping. The labels (ASD or TD) of the training data set were randomly shuffled to simulate a completely uninformative data set, and the accuracy of a classifier trained on the artificial data was noted. The process was repeated 99 times.

**Fig 4:**
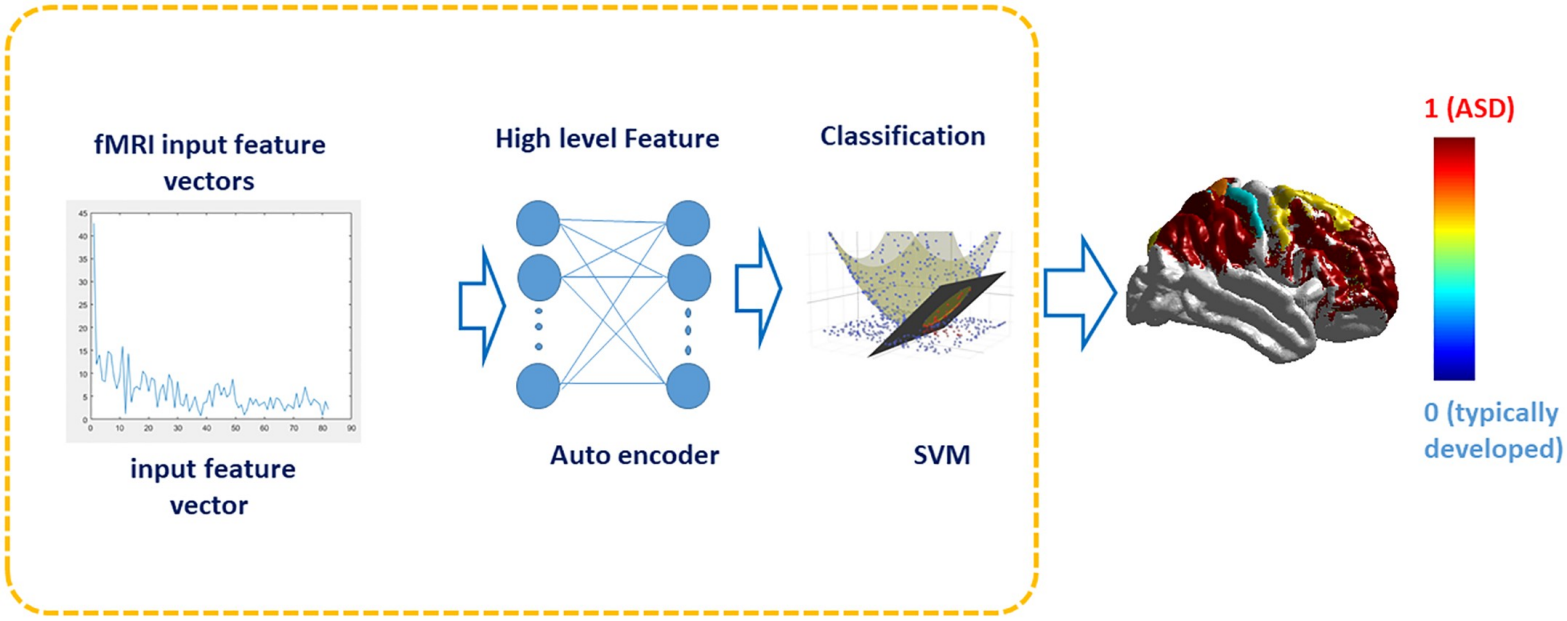
The diagnosis pipeline, where PSD is fed to SAE to extract higher level features. The extracted features are fed to SVM. The classification output is a global diagnosis decision, indicating whether the subject is autistic or healthy controls; in addition a personalized color coded map indicating how much each area in the subject is implicated by autism impairments.

For any new unseen subject, the output of this diagnosis system both makes a global decision indicating whether the subject is autistic or healthy controls, and it generates a vector of area membership scores indicating how much every area is implicated by autism related impairments. Fig 4 illustrates the diagnosis input, pipeline, and output.

To test the system performance, two different validation techniques are used: (i) cross validation, where 2-folds, 4-folds, 10-folds, and leave one subject out (LOSO) are used, and (ii) hold-out testing by data partitioning to training dataset (60% of the data), validation dataset (15% of the data) and testing dataset (25% of the data). For each of the validation techniques used, accuracy, sensitivity, specificity and area under ROC curve are calculated. To show the effect of hyper-parameters on model accuracy, the supplementary materials—S2 Table is now uploaded that shows the accuracies per component for different combinations of hyper parameters.

Also, to check the scalability and extendibility of the diagnostic system, positive and negative predictive values (PPV and NPV) are estimated:

$$\begin{array}{c} PPV=\frac{sens.prev}{sens.prev+(1-spec).(1-prev)} \end{array}$$ (3)

$$\begin{array}{c} NPV=\frac{spec.(1-prev)}{\left(1-sens).prev+spec.(1-prev\right)} \end{array}$$ (4)

where *Sens* and *Spec* are the sensitivity and specificity of the classifier, respectively, and Prev is the prevalence or prior probability of a diagnosis of ASD. In this study, two different prevalence values are used. The first prevalence value is 1 out of 68, which is the ASD ratio in the United States population [57]. The second prevalence value is 18.7%, which indicates the autism percent among a high-risk population, where an older sibling has been previously diagnosed with ASD [58].

## Results

### Subjects’ demographics, cognitive and behavioral data

ASD and TD subgroups were well-matched with respect to gender and age. Out of 123 ASD subjects, 56 were female (45.5%), while 85 of the 160 TD subjects were female 53.1%). The gender imbalance was statistically insignificant (*χ*^2^ = 0.05, *p* = 0.82). The mean age of ASD subjects was 13.1 years, while the mean age was 12.9 years for the TD group. Again, the difference was statistically insignificant (*t* = 0.302, *p* = 0.763). The groups were less well matched with respect to IQ, although the differences in mean scores were less than one standard deviation (Table 2). It was noted that 38 of those with ASD were prescribed medication for behavioral concerns, 19 used prescription medication for reasons other than behavioral, 20 used over-the-counter medication, and 29 took dietary supplements. Medication status data is incomplete, with missing data for six ASD individuals and no available data for any of the healthy controls subgroup. Those diagnosed with ASD presented with a wide range of severity on the ADOS (Table 2).

**Table 2:** Summary of study cohort. DAS-II GCA: General cognitive ability. ADOS S.A.: Social affect, R.R.B. Repetitive and restricted behaviors and interests. p-values are from an unpaired t-test of ASD and TD groups having equal mean value.

	ASD (N = 123)		TD (N = 160)		*p*-Value
**Age(y)**	Mean	S.d.	Mean	S.d.	0.675
Male (N = 152)	12.87	3	13.0	2.8	
Female (N = 131)	13.1	2.5	12.9	3.09	
**DAS-II**	Mean	S.d.	Mean	S.d.	*p*-value
GCA	102	21.2	111	15.6	0.001
Verbal	103	22.6	112	15.2	0.001
Nonverbal	101	19.2	109	16.2	0.003
Spatial	100	17.5	107	13.8	0.012
**ADOS**	Median	Range			
S.A.	8.8	0–19			
R.R.B.	2.4	0–6			
Cumulative	11.1	1–24			

### Global and personalized diagnosis results

**Fig 5:**
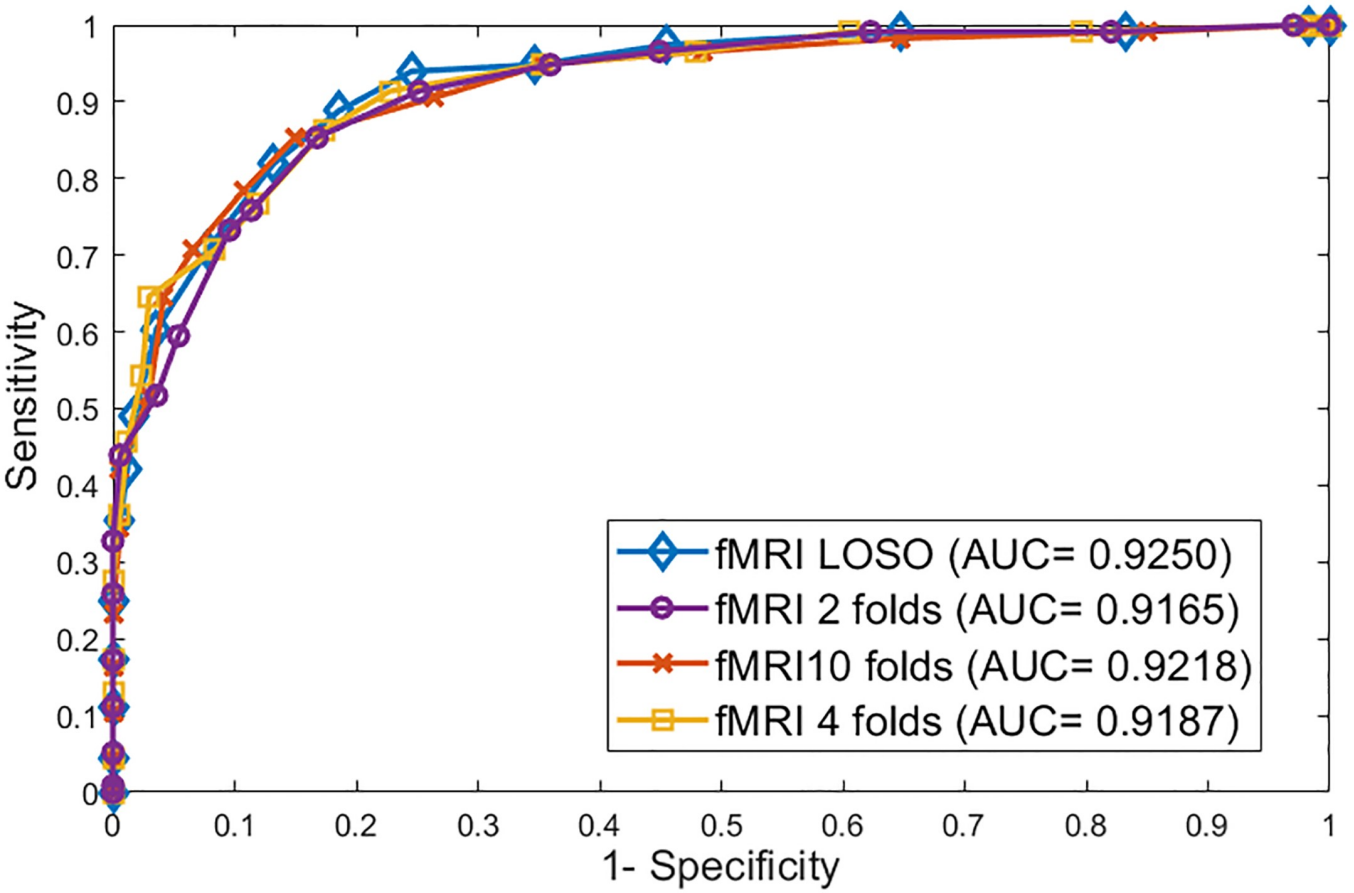
The ROC curves for 2-folds, 4-folds, 10-folds, and leave one subject out cross validation experiments.

For the 2-folds cross validation, the accuracy is 0.84, sensitivity is 0.88, specificity is 0.81, and AUC is 0.9165. For the 4-folds, the four metrics values are 0.88, 0.90, 0.87 and 0.9187 respectively, while for the 10-folds they are 0.91, 0.92, 0.88 and 0.9218. And finally for LOSO, they are 0.92, 0.93, 0.89 and 0.9250, respectively. Table 3 summarizes the four metrics used for every cross validation technique. Also Fig 5 shows the ROC curves for the 4 cross validation experiments.

**Table 3:** Accuracy, sensitivity, specificity, and AUC for 2-folds, 4-folds, 10-folds, and LOSO cross validation experiments.

	2-folds	4-folds	10-folds	LOSO
Accuracy	0.84	0.88	0.91	0.92
Sensitivity	0.88	0.90	0.92	0.93
Specificity	0.81	0.87	0.88	0.89
AUC	0.9165	0.9187	0.9218	0.9250

To make sure that the system is robust enough, each of the k-fold cross validations is repeated 100 times and a summary statistic of the accuracy is reported in Table 4. Minimum accuracy, maximum accuracy, mean accuracy, and accuracy standard deviation are reported. They show homogeneity in the results over 100 runs which gives a good indication of the system robustness. Also, the bootstrap p-value for accuracy of classification was estimated to be 0.01.

**Table 4:** The summary statistics of accuracy after running 2-folds, 4-folds, 10-folds, and LOSO 100 times.

	2-folds	4-folds	10-folds	LOSO
Min accuracy	0.8	0.84	0.85	0.87
Max accuracy	0.87	0.92	0.92	0.94
Mean accuracy	0.83	0.87	0.88	0.91
Accuracy standard deviation	2.2	3.1	2.5	3.2

In addition to using the cross validation technique for system evaluation, we are also using hold-out testing. In this technique, data is divided into 3 partitions: training, validation, and testing. This experiment aims to assess the system generalization ability and it ensure that the obtained results are robust and reproducible. The obtained accuracy from this experiment is 0.91, sensitivity is 0.88, and specificity is 0.92.

To highlight the effect of using both SAE and SVM, 12 combinations obtained from using 3 dimensionality reduction techniques and 4 different classifiers reported in Table 5. The three dimensionality reduction techniques are: (i) SAE, (ii) PCA, and (iii) Kernel PCA The four algorithms used are: (i) SVM, (ii) random forest, (iii) logistic regression, and (iv) neural network. For all the used algorithms, the hyper-parameters are also selected using a grid searching algorithm. All of the 16 combinations are reported using hold-out testing techniques and accuracies are reported with respect to the testing dataset.

**Table 5:** A comparison of accuracies obtained using 4 different classifiers and 3 different dimensionality reduction techniques, in addition to the accuracies obtained when PSDs are fed to the classifiers directly are shown. All these accuracies are reported using hold-out testing technique. All classifiers and dimensionality reduction technique hyper-parameters are fine tuned using the grid searching algorithm. The highest accuracy obtained is 93%. It is obtained using SAE followed by SVM with RBF kernel.

	PCA	Kernel PCA	SAE	No dimensionality reduction (PSD is used directly)
RBF SVM	0.83	0.84	**0.93**	0.84
Random Forest	0.76	0.83	0.82	0.81
Logistic Regression	0.83	0.81	0.81	0.79
Neural Network	0.82	0.86	0.91	0.82

The positive and negative predictive values are also calculated using the two prevalence values mentioned in the methodology section. The PPV and NPV indicate the probability of match between the actual diagnosis and the system output diagnosis when applying the system to a population with the prevalence used in PPV and NPV calculations. For the general prevalence, the PPV is 0.19 and the NPV is 0.91, while for high-risk prevalence, the PPV is 0.79 and the NPV is 0.9. Table 6 summarizes the PPV and NPV for the two prevalence values used.

**Table 6:** The PPV and NPV reported when using two different prevalence values, one for the general population in the USA (1/68), and the other for the high-risk population (18.7%).

	Prevalence 1 (1/68)	Prevalence 2 (0.187)
PPV	0.19	0.79
NPV	0.91	0.9

**Fig 6:**
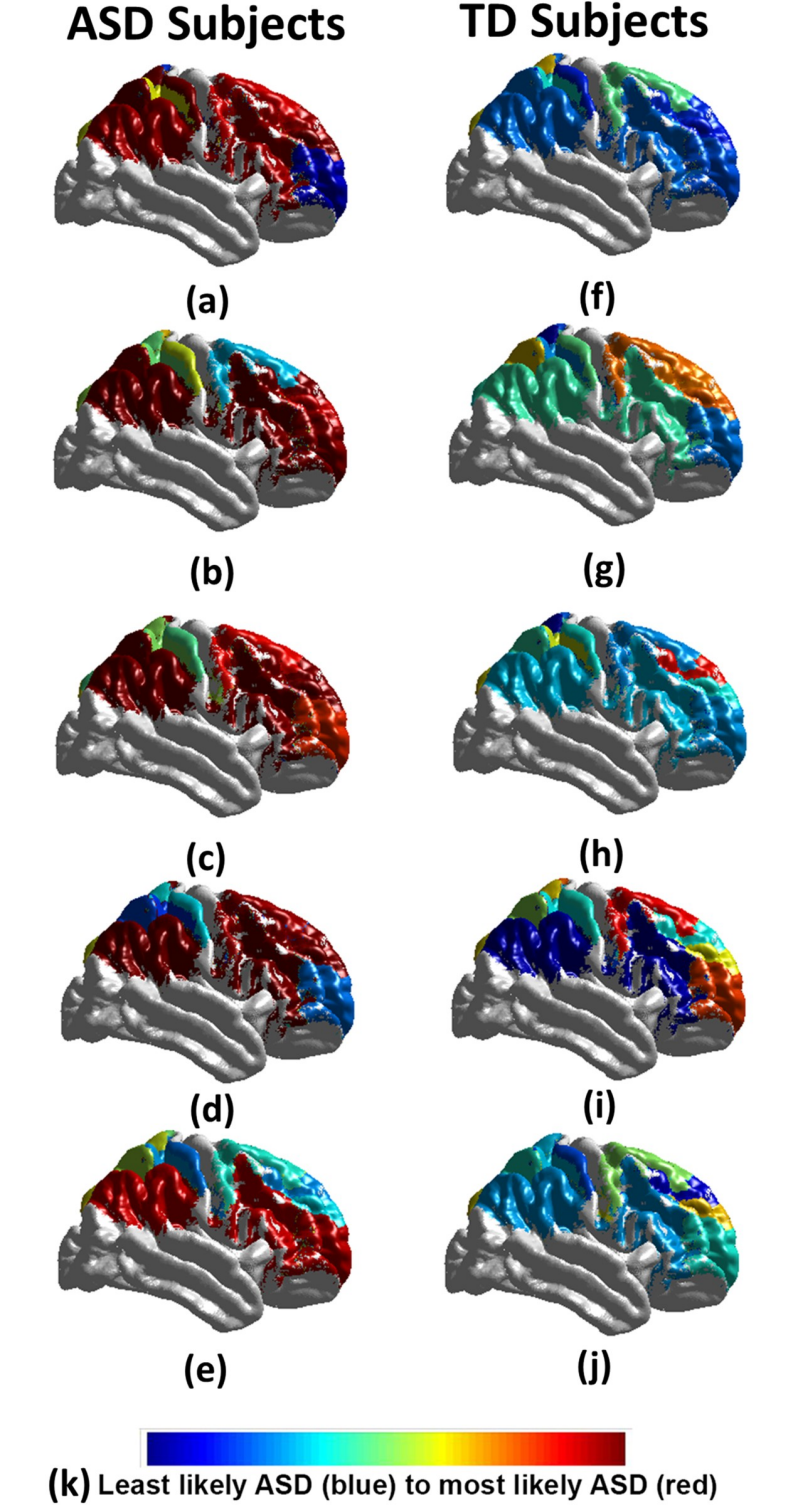
The personalized results of 10 autistic subjects (a-e), 10 healthy controls (f-j); (k) represents the color code used, where blue is the least belonging to autistic class, and red is the most belonging to the autistic class. It is obvious that autistic subjects have more impacted areas than the healthy controls.

**Fig 7:**
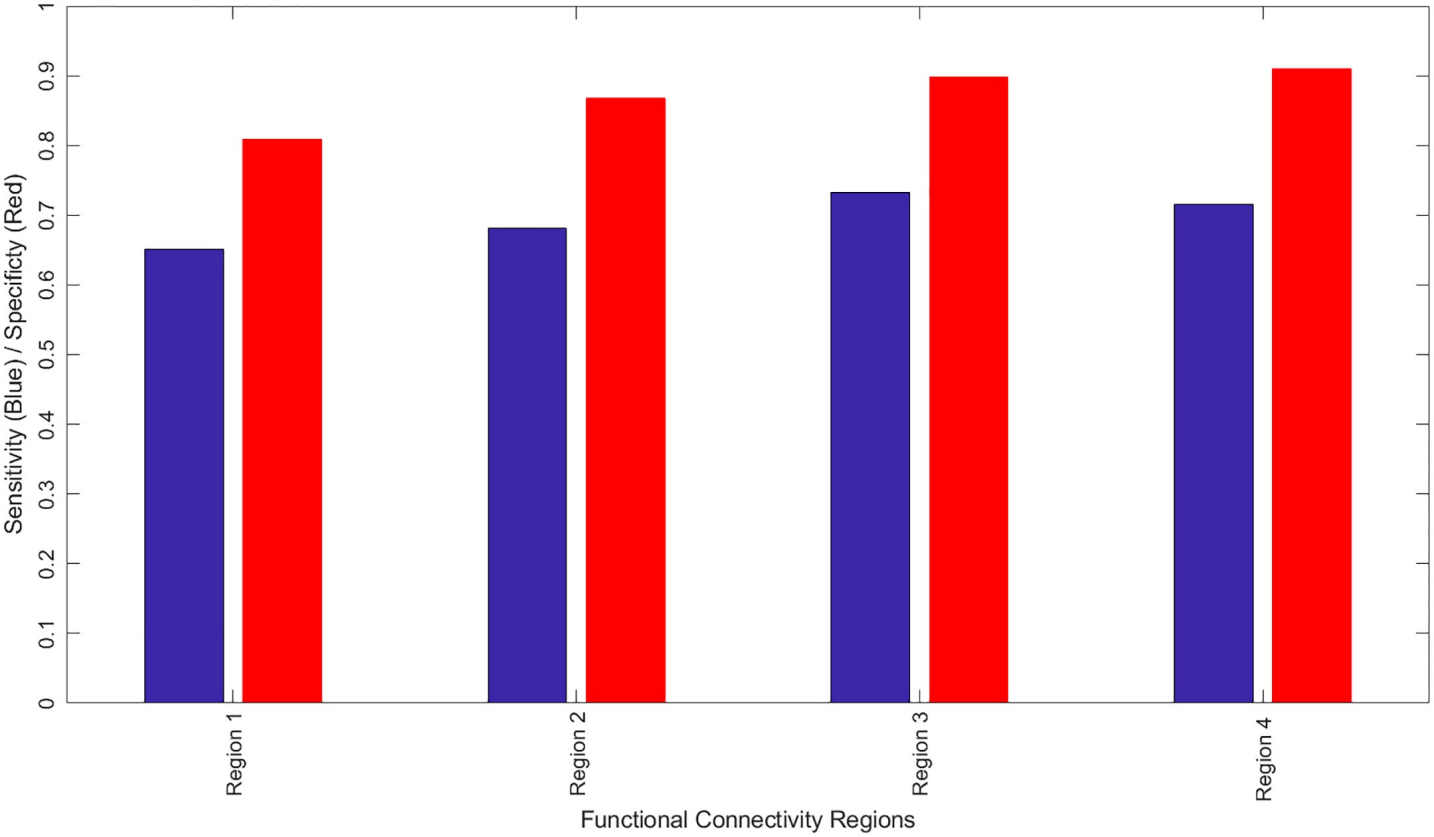
The regions in the fMRI experiment that have both sensitivity and specificity greater than 0.65, as well as highly corerleted with ADOS reports when using 4-fold cross validation. Region 1: BA24, 32, 34 R and L, anterior cingulate gyrus, BA9/10, R and L medial frontal gyrus, BA9/10, R middle frontal gyrus, BA8, right superior frontal gyrus, right caudate nucleus; Region 2: BA22 superior temporal gyrus L≥R, BA19 middle temporal gyrus L≥R, BA39 middle temporal gyrus L≥R; Region 3: BA8/BA9/BA10, Left and Right superior frontal gyrus, BA9/BA10, L and R medial frontal gyrus, BA9/BA10, Right middle frontal gyrus, and Region 4: BA10, Left middle frontal gyrus, BA10 Left superior frontal gyrus.

In addition to reporting the global diagnosis result, a detailed report is generated showing a personalized local diagnosis for every subject. Using this report a color coded brain map is generated to show areas most affected with autism related impairments. Fig 6 shows 10 samples of the color coded brain maps with the associated color code used. To identify the functional areas that are highly related to autism diagnosis between the two groups, the sensitivity and specificity of each individual area are calculated, where the area sensitivity and specificity are obtained using the true negative and true positive rates of the diagnosis when using this separate area alone. A bar graph is provided in Fig 7 to illustrate the most significant regions with both sensitivity and specificity above an empirical threshold of 0.65 obtained. They are highly correlated with behavioral reports when using 4-fold cross validation. The full personalized results of the all subjects used with the membership scores of the significant areas to the autism class is in supplementary materials—S1 Table.

**Fig 8:**
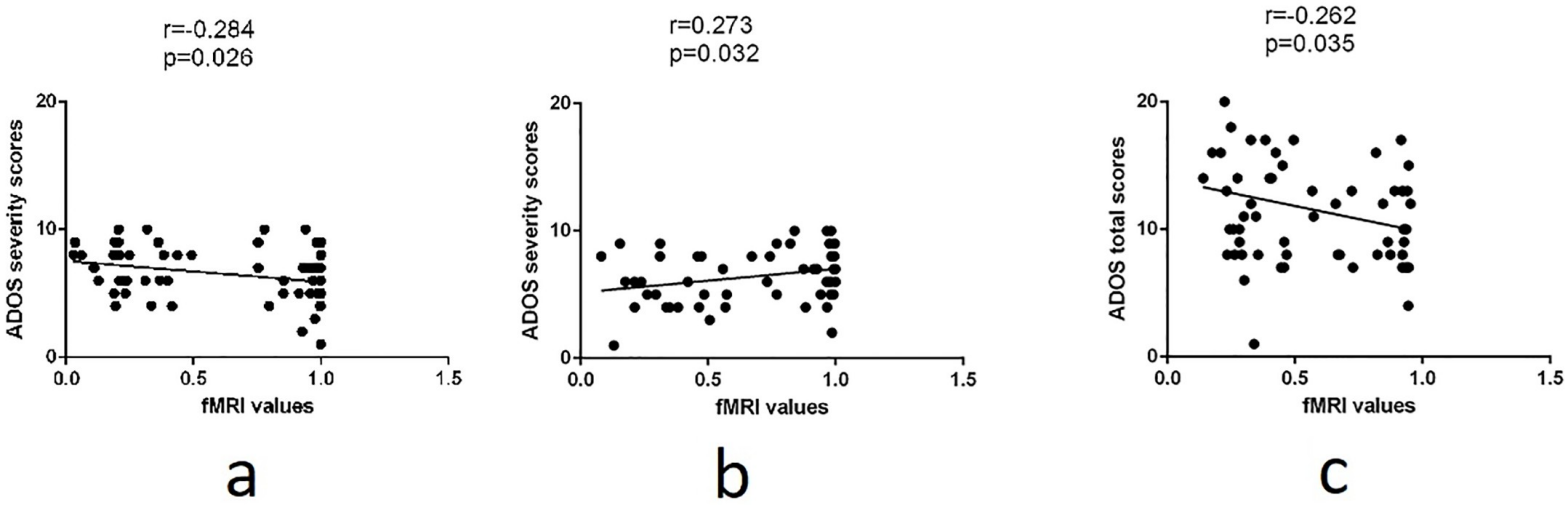
The correlation between obtained ADOS scores (either severity or total) from the personalized diagnosis and (a) Fpl, (b) TPJb, and (c) cluster 4.

To cross validate the relevance of these regions to ASD, each brain region was correlated with the Total ADOS score and ADOS severity score. The Pearson correlation coefficient varies modestly from -0.28 to 0.27 for Brodmann area/brain regions involved in neurocircuits previously implicated in ASD (Table 7 and Fig 8).

**Table 7:** Mapping between ADOS subscores, Research Domain Criteria (RDoC) neurocircuits, and functional connectivity networks. Anatomical (Brodmann) areas overlapping functional networks are given in parentheses.

Component	RDoC Defined Neurocircuit	Anatomical correspondence
Restricted Interest/Repetitive Behaviors	Reward Learning	Fpl, Cluster 4 (BA10)
	Habit	Fpl, Cluster 4 (BA10)
Attention	Ventral Attention System	Fpl, Cluster 4 (BA10), TPJb (BA 39-40, 22)
Language	Receptive	TPJb (BA 39-40, 22)
Social	Affiliation and Attachment	Fpl, Cluster 4 (BA10)
Social	Understanding the Mental States of Others	Fpl, Cluster 4 (BA10), TPJb (BA 39-40, 22)
Executive Function	Working Memory	TPJb (BA39-40)

## Discussion

Over the last few years, remarkable progress in MRI research has allowed the prospective identification of infants with ASD at 24 months based on structural MRI or fMRI features [59]. The methodology presented in this paper using machine learning algorithms allows the identification of ASD vs healthy controls children and teenagers who are age 8-18 years of age with high accuracy, sensitivity, and specificity (Tables 3–6; Figs 6 and 7). In this study. we introduced an automated autism diagnosis system that uses resting state fMRI to localize the altered connectivity patterns for each subject. In addition, the system showed very promising generalization to all populations and certainly in the high-risk population (Table 4). The sample used in this study is well matched in terms of age and gender distribution (Table 2). Although the IQ mean scores are statistically significant, these data are less than one standard deviation apart. In addition, the full range of IQs in healthy controls is fully included inside that of the ASD group.

In general, ASD is thought to be a developmental disconnection syndrome with local functional hyperconnectivity and long range functional underconnectivity [60]. Both task based and resting state fMRI demonstrate a clear separation via unique BOLD patterns between ASD and healthy control children, teenagers, and adults. fMRI is dependent upon a number of maturation factors including gene expression, numbers of synapses, cell numbers, synaptic pruning, myelination, etc., which may limit the developmental context of the signal interpretation [61, 62]. Even in separation of these groups (ASD vs healthy controls), one must remember the developmental context. In high risk infants, R-fMRI networks at 6 months of age correctly predicted those with an ASD diagnosis at 24 months with a sensitivity of 82% (9 out of 11 infants with ASD) and a specificity of 100% (48/48 of those infants without ASD) [59]. However, very few R-fMRI networks were correlated with social communication and cognitive ability in high-risk infants, but many more networks were correlated with repetitive behaviors (self injury, stereotypes, sameness, ritualistic behaviors, compulsions). The interpretation suggests a developmental context since striatal and brainstem neural networks tend to mature earlier than cortically based networks. Alternatively, R-fMRI data could correlate more with ASD core symptoms, and anatomical MRI could be more closely correlated with cortically based symptoms (sensory problems, language impairment, etc.). In the older population, the current functional MRI algorithm identifies similar regions with altered connectivity previously noted in ASD including the pre-motor/supplementary motor cortex, dorsal lateral and medial prefrontal cortex, sensorimotor cortex/superior parietal lobule/supramarginal gyrus, and regions involved in language (angular gyrus, supramarginal gyrus [63]) being predictive of ASD with a high accuracy, sensitivity, and specificity (Tables 3–6 and Figs 6 and 7) across many models.

The total ADOS score and the ADOS severity score modestly correlate with brain regions (lateral frontopolar region and temporal parietal junction) in deficit cognitive circuits previously implicated in ASD (Table 7, Fig 8) according to Research Domain Criteria (RDoC: https://www.nimh.nih.gov/research-priorities/rdoc/constructs). These deficits could impact restricted interest/repetitive behaviors, attention, social, language, and executive function.

This fMRI algorithm may be more predictive in those of high risk ASD families than in the general population (Table 6). Previous R-fMRI studies have identified some of these regions such as parts of the Default Mode Network, including medial prefrontal cortex and the angular gyri, and interhemispheric connectivity networks (sensorimotor cortex and superior parietal lobule) with reduced connectivity in ASD [63]. Thus, the current data presented suggest the algorithms, especially when combined in a multi-modal approach, have the potential to identify diagnostic category and clear brain regions involved in classical neural circuits previously implicated in ASD. In addition to providing a highly accurate prediction of a subject to have ASD, the proposed system provides a complete map explaining what areas are affected, and to what extent they are affected. (Fig 6 and supplementary materials S1 Table). To the best of our knowledge, this detailed report gains its importance from being the first work that is concerned with localizing impairments for each subject’s brain. The utility of this approach not only identifies those with ASD but might be of more help ascertaining specific impairments and thus, quite useful from the clinical point of view.

## Limitations of the approach

Neuroimaging is an attractive intermediary to bridge the gap between genes, environment, and well-defined behavioral phenotypes such as ASD. The idea is to obtain a clinically relevant scan which one can then more closely relate to the neurobiological pathway of risk genes, other biofactors, and/or environmental factors on an individual level. The drawback of the current data and MRI-based methods is defining the developmental trajectory, impact of age/gender, development of clinically applicable techniques for scanning across ages, and the relationships to current clinical psychological methods to diagnose ASD. The current data may only be applicable to high functioning and older ASD patients but may be insensitive at younger ages (Table 2). The current sample size which identified areas implicated in younger children and infants as being predictive of ASD suggest a scalability of this approach to larger more heterogeneous populations. The use of R-fMRI data represents a particular challenge since the field is underdeveloped. The diversity of the subject pool (age/gender), design of the resting state scan, and the preprocessing/methods of analyses are still variables under study. Most importantly, there is a lack of longitudinal data defining normal functional connectivity in infancy through 8 years of age, and thus defining the abnormal developmental trajectory in ASD is difficult under 8 years of age [63]. The analysis in this study was performed using FSL package, to eliminate any limitations from the package and to gain more flexibility to try different recent and up to date approaches at each analysis phase, a home developed package is being developed to be used in future studies. It is believed that more generalization and feasibility of the system could be studied by increasing the number of subjects and the intra-variability between subjects, including age group, multiple sites and multiple scanners data and other factors. This could be achieved by integrating multiple sites and multiple data sources in the dataset used.

## Conclusion and future work

The advancement of new research technologies, including sMRI, fMRI, DTI, and genomics has made significant inroads into the potential identification of biomarkers for ASD. Despite significant efforts, smaller studies have made it difficult to generalize findings to larger more heterogeneous populations [62]. This study demonstrates that data points from R-fMRI and machine learning algorithms could refine diagnostic accuracy, with the potential to predict clinical phenotypes, and the potential to develop better individualized treatments. Specific affected networks could be a biomarker for responses to specific types of behavioral interventions (i.e., individual psychotherapy, occupational therapy for sensory impairments, social skills training, etc.) or drug trials (i.e., selective serotonin uptake inhibitors VS. antipsychotic medications, etc.). In addition, the fMRI data could identify more genetically homogeneous groups in which specific neuropathological processes—such as decreased axonal pruning leading to increased mini-column width and altered synaptic connectivity—are common in specific networks of those with similar defects in axonal or synaptic gene function [5]. Future research should focus further on using big data technology to combine multiple datasets from larger populations to better delineate clinically relevant neurobiological pathways and determine response to therapies in ASD. In addition, integrating information from multiple data sources such as behavioral reports and genetic profiles to get more insight about areas of interest observed on each individual subject would be helpful. Future research should also focus on studying the preprocessing steps individually in a more detailed manner as they are reported to have an important role in the diagnosis. For example, the patient’s head movement may cause significant noise and affect the fMRI measures, causing classification bias [64], [65]. The motion correction in this study was done using the MCFLIRT algorithm [38] which yielded good results, but more recent motion correction algorithms might be more efficient in the future work. The next phase of this study should focus on including multiple site data from different datasets (ABIDE, for example) to study system robustness and generalization ability.

## Supporting information

S1 Table Personalized result of all 283 subjects used. In this table, a score between 0 and 1 is used to define the membership score of each area to the autism class, with 0 being normal and 1 being the highest in the autism class membership. The scores in this table are used to generate the color coded visualization in Fig 6.
(XLSX)

S2 Table Sets of hyper-paramters used for both autoencoder and SVM for each individual area during the grid search Fig 7. (XLSX)

S1 Fig The selection of hyper-parameters in a K-fold cross validation using grid search algorithm.2. (DOCX)
