# Supplementary material for: Using resting state functional MRI to build a personalized autism diagnosis system
Source: PLoS One. 2018 Oct 31;13(10):e0206351. doi: 10.1371/journal.pone.0206351 (PMC6209234; doi:10.1371/journal.pone.0206351)

**S1 Fig**

To select the hyper-parameters in a K-fold cross validation using grid search algorithm, the following Pseudo code is used:


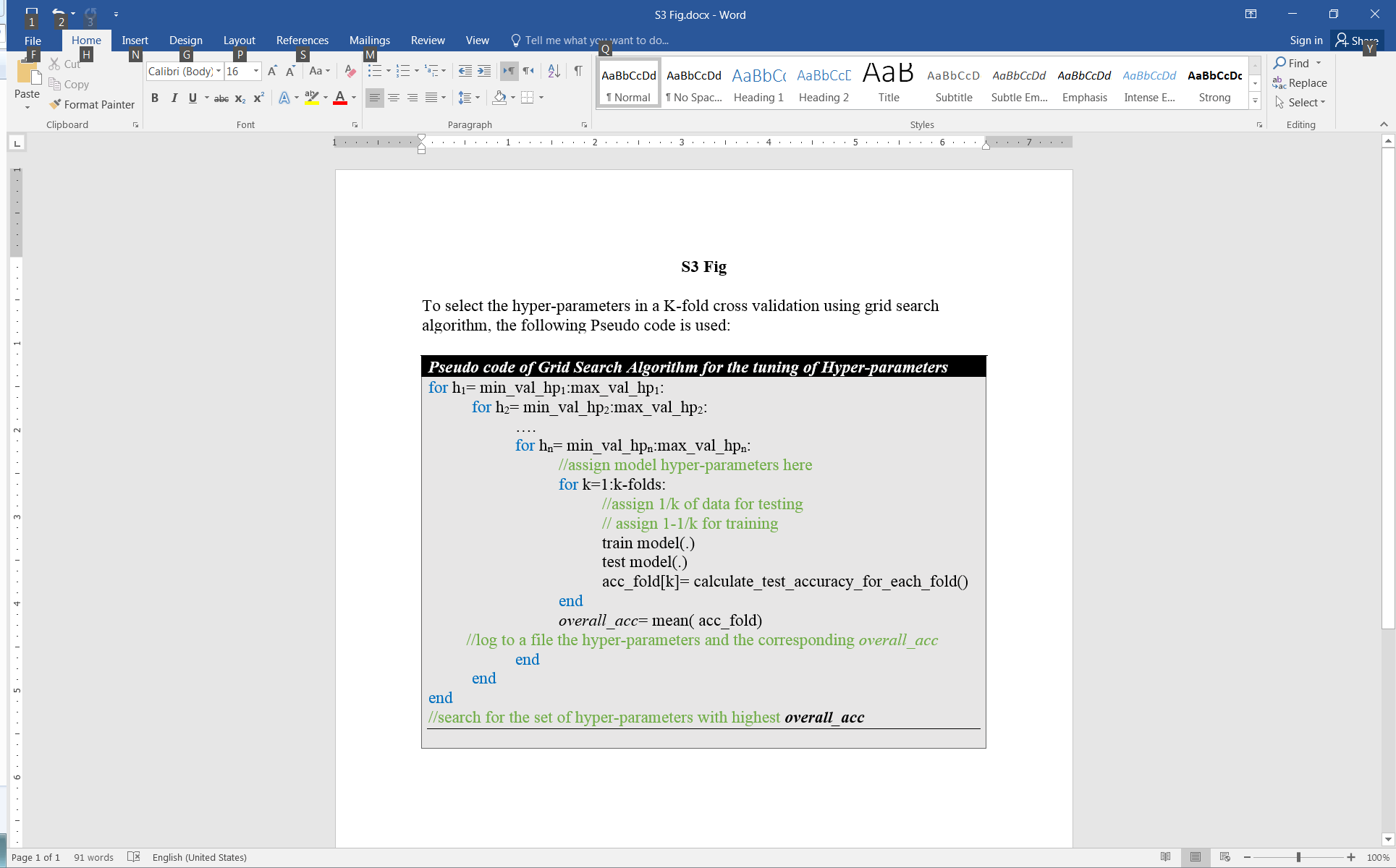

Supplement: S1 Fig — (DOCX) [file pone.0206351.s003.docx]
